# Comparative In Vitro Characterization of the Nutritional and Functional Properties of Commercially Available Black Soldier Fly Larvae Meal and Soybean Meal

**DOI:** 10.3390/ani16203282

**Published:** 2026-10-18

**Authors:** Ezgi Şenses, Berkay Erdoğmuş, Arda Çelik

**Affiliations:** Department of Animal Science, Faculty of Agriculture, Bursa Uludağ University, Bursa 16059, Turkey; ezgisenses@uludag.edu.tr (E.Ş.); berkaye@uludag.edu.tr (B.E.)

**Keywords:** black soldier fly larvae, *Hermetia illucens*, defatted, amino acids, fatty acids, phenolic compounds, antioxidant capacity, poultry nutrition

## Abstract

This study provided an integrated comparative characterization of the nutritional and in vitro functional characteristics of commercially available defatted black soldier fly larvae meal (DF-BSFLM), full-fat black soldier fly larvae meal (FF-BSFLM), and soybean meal (SBM). DF-BSFLM had the highest crude protein concentration (55.70%), whereas in vitro crude protein digestibility was 72.87%, 75.26%, and 88.75% for DF-BSFLM, FF-BSFLM, and SBM, respectively. FF-BSFLM had the highest ether extract concentration (37.09%) and predicted apparent metabolizable energy (AME) value (21.92 MJ/kg), followed by DF-BSFLM (15.86 MJ/kg) and SBM (10.98 MJ/kg). SBM had higher concentrations of several essential amino acids, including leucine, isoleucine, arginine, and phenylalanine, whereas DF-BSFLM had higher concentrations of methionine and valine. FF-BSFLM had the highest saturated fatty acid content (57.57%) and lauric acid concentration (18.79%), whereas SBM had the highest polyunsaturated fatty acid content (51.71%). DF-BSFLM had the highest total Folin–Ciocalteu-reactive content (64.6 mg GAE/g) and DPPH radical-scavenging activity (48.2 mg TE/g), compared with 40.9 mg TE/g for SBM and 27.7 mg TE/g for FF-BSFLM. Overall, the tested ingredients exhibited distinct nutritional and in vitro functional profiles. These findings provide a product-level comparison of commercially available BSFLM products with SBM. Because the FF- and DF-BSFLM products were obtained commercially rather than generated from a common batch under controlled conditions, the observed differences cannot be attributed exclusively to defatting. Furthermore, each ingredient was represented by a single commercial batch, and the analytical replicates do not establish product-level variability. Further studies involving multiple independent commercial batches and controlled in vivo trials are needed to assess product variability and evaluate nutrient bioavailability, standardized ileal amino acid digestibility, energy utilization, and physiological responses in poultry.

## Simple Summary

Sustainable protein sources are increasingly needed to support the development of poultry production. Among the alternatives being investigated, black soldier fly (*Hermetia illucens*, BSF) larvae offer potential as an insect-derived feed ingredient. This study characterized commercially available full-fat and defatted BSF larvae meals in terms of their nutritional composition and in vitro functional properties and compared them with soybean meal (SBM), a conventional protein ingredient. The defatted BSF larvae meal had higher total phenolic content and in vitro DPPH radical-scavenging activity, whereas the full-fat meal had higher ether extract concentration and predicted apparent metabolizable energy and was characterized particularly by its high lauric acid content. SBM exhibited a more unsaturated fatty acid profile and contained higher amounts of several essential amino acids. These findings demonstrate distinct nutritional and in vitro characteristics among the commercially available ingredients evaluated in this study. Because the BSFLM products originated from different commercial sources, the observed differences cannot be attributed specifically to defatting. Further controlled and in vivo studies are needed to determine nutrient bioavailability, energy utilization, and physiological responses to these products in poultry.

## 1. Introduction

Alternative feed ingredients that may reduce reliance on conventional protein sources have received increasing attention because of the growing demand for sustainable and nutritionally efficient feed resources [1,2]. Due to its high protein content, favorable amino acid profile, and widespread availability, soybean meal (SBM) remains one of the principal protein sources used in animal nutrition. However, the presence of anti-nutritional factors may affect nutrient utilization, while soybean production is associated with environmental concerns [3,4]. Consequently, there is growing interest in alternative protein ingredients that combine desirable nutritional characteristics with additional functional properties [5].

Insects have attracted considerable attention as alternative protein sources because of their high nutrient density, efficient feed conversion, and ability to transform organic substrates into biomass. Among insect species, the black soldier fly (*Hermetia illucens*; BSF) is particularly promising for animal feed applications because its larvae can utilize a wide range of organic substrates and convert them into nutrient-rich biomass [5]. Recent studies have shown that the composition and nutritional quality of BSF larvae can be influenced by the type of rearing substrate, with differences in substrate composition affecting larval growth, nutrient composition, and bioconversion efficiency [6,7]. The chemical composition of BSF larvae can also vary according to developmental stage, rearing conditions, and processing procedures, contributing to considerable variation among BSF-derived feed ingredients [5,6]. In particular, defatting can substantially modify the nutritional profile of BSF larvae by reducing the lipid fraction and increasing the relative concentration of crude protein [8,9]. Recent research has further demonstrated that the extent of lipid removal can influence the resulting energy value, nutrient digestibility, and overall nutritional characteristics of BSF meals [9,10]. Therefore, characterization of commercially available BSF products is important because differences in processing history and product composition may affect their potential nutritional value as alternative feed ingredients.

Black soldier fly larvae meal (BSFLM) contains appreciable amounts of protein, essential amino acids, lipids, and minerals, supporting its potential use as an alternative feed ingredient [5,11,12]. Previous studies have characterized the nutritional composition, energy value, digestibility, and fatty acid profiles of BSFLM, including differences associated with its processing form [8,9,10,13,14]. However, findings obtained under specific experimental conditions do not necessarily represent the characteristics of commercially available products evaluated as feed ingredients. In particular, comparisons between commercially available full-fat and defatted BSFLM products require careful interpretation because differences between products may reflect not only lipid removal but also variation in raw materials, rearing conditions, developmental stage, and processing history. In the present study, FF-BSFLM and DF-BSFLM were obtained from the same commercial supplier but represented commercially available products rather than experimentally generated fractions derived from a common batch. Although both products were obtained from the same supplier, this does not establish that they originated from the same rearing substrate, larval population, production conditions, or processing history. Accordingly, their comparison is interpreted as a product-level characterization rather than an experimental evaluation of the effects of defatting. Although previous research has addressed individual nutritional characteristics of BSFLM, a comparative characterization encompassing proximate composition, amino acid and fatty acid profiles; in vitro crude protein digestibility; and predicted apparent metabolizable energy (together with in vitro functional measurements) can provide complementary information for evaluating commercial BSFLM products relative to soybean meal (SBM), an established plant-based protein source. The value of this approach lies not in the number of parameters measured alone but in the joint characterization of nutritional and functional attributes within a common analytical framework.

Beyond its nutritional composition, BSFLM has attracted interest because of its potential functional and bioactive properties. Rearing substrate and processing conditions have been reported to influence phenolic content and antioxidant-related characteristics of BSF-derived products [15]. Total phenolic content (TPC) and DPPH radical-scavenging activity provide complementary in vitro measurements of Folin–Ciocalteu-reactive content and radical-scavenging activity, respectively. However, the Folin–Ciocalteu assay is not specific to phenolic compounds, and DPPH activity reflects the radical-scavenging response under the assay conditions rather than demonstrating physiological antioxidant effects. Considering these measurements alongside nutritional composition, digestibility, and fatty acid profiles allows the commercial products to be characterized beyond their conventional nutrient composition while avoiding assumptions about their biological effects. Accordingly, the present study aimed to provide an integrated in vitro characterization of commercially available FF-BSFLM and DF-BSFLM in comparison with SBM. Proximate composition, starch and total sugar concentrations; in vitro crude protein digestibility; amino acid and fatty acid profiles; predicted apparent metabolizable energy; TPC; and DPPH radical-scavenging activity were evaluated within the same analytical framework. The study was designed to describe the nutritional and in vitro functional profiles of the specific commercial products evaluated, rather than to establish the causal effects of defatting or to infer differences among commercial batches.

## 2. Materials and Methods

### 2.1. Experimental Materials

Three commercially available protein ingredients were evaluated in the study; DF-BSFLM, FF-BSFLM SBM. The DF-BSFLM and FF-BSFLM samples were commercially obtained from Sino Crown (Qingdao, Shandong, China), whereas the SBM was supplied by a local feed company in Balikesir, Türkiye. For each ingredient, a single commercial batch was used. Each ingredient was dried, ground to a uniform particle size, thoroughly homogenized, and divided into five analytical test portions for subsequent analyses. Thus, the five test portions represented analytical replicates from the same homogenized commercial batch for each ingredient. The prepared samples were transferred into airtight containers and stored under appropriate conditions until further analysis.

### 2.2. Chemical Composition Determination and Predicted Apparent Metabolizable Energy Calculation

The proximate composition of the experimental feed ingredients was determined according to the methods of the Association of Official Analytical Chemists (AOAC) [16]. The contents of dry matter (DM), crude ash (CA), crude protein (CP), ether extract (EE), starch (S), and total sugar (TS) were determined using standard analytical procedures. For each ingredient, five analytical test portions were obtained from the thoroughly homogenized material and then analyzed. All chemical composition results were expressed on a DM basis.

For DM determination, samples were dried in a hot-air oven at 100 °C until a constant weight was obtained. Moisture content was calculated from the difference between the initial and final sample weights and expressed as a percentage of the initial sample weight [16].

The CA content was determined with minor modifications to the AOAC procedure [16]. Each sample (5.0 g) was accurately weighed into a preheated, preweighed crucible and incinerated in a muffle furnace at 600 °C for approximately 5 h. After cooling in a desiccator, the crucibles were reweighed, and the CA content was calculated from the difference in weight.

The CP content was determined by the Kjeldahl method according to AOAC Official Method 984.13 [16], using a [manufacturer, model] Kjeldahl digestion and distillation system (Gerhard Analytical Systems, Yellow Springs, OH, USA). The nitrogen concentration was multiplied by a conversion factor of 6.25 to calculate CP content. The EE content was determined according to AOAC Official Method 954.02 [16]. Samples were hydrolyzed with hydrochloric acid and then transferred to Mojonnier flasks. Lipids were extracted using a Soxhlet apparatus with a mixture of hexane and ethyl ether. The extracts were passed through anhydrous sodium sulfate to remove residual moisture, then evaporated to dryness and weighed. Ether extract content was expressed as a percentage of the initial sample weight and subsequently converted to a DM basis.

The S content of the experimental feed ingredients was determined using the Ewers polarimetric method specified in Commission Regulation (EC) No 152/2009 for the determination of starch in feed [17]. Then, 2.5 g of the ground sample was weighed into a flask, treated with hydrochloric acid, and subjected to hydrolysis. The solution was subsequently clarified and filtered, and the filtrate was made up to the required volume with distilled water. The optical rotation (P) was measured using a polarimeter equipped with a 200 mm observation tube (Pol-200 semiautomatic polarimeter, Bante, Sugar Land, TX, USA). A second determination was performed to account for the optical rotation contributed by substances soluble in 40% (*v*/*v*) ethanol. For this determination, 5 g of the sample was extracted with 40% (*v*/*v*) ethanol. Following filtration, an aliquot corresponding to 2.5 g of the original sample was transferred to a flask, treated with hydrochloric acid, heated in a boiling water bath for 15 min, cooled, clarified, and made up to volume with distilled water. After filtration, the optical rotation (P′) was measured under the same conditions.

The S content was calculated from the difference between the total optical rotation and the optical rotation attributable to substances soluble in 40% (*v*/*v*) ethanol. For samples containing other starch types or mixtures, a specific optical rotation of 184.0° was used, as specified for starch mixtures in compound feed. The starch content was calculated using the following equation:

S (%) = (2000 × (P − P′))/184.0

where P is the optical rotation measured for the total starch fraction (degrees), P′ is the optical rotation attributable to substances soluble in 40% (*v*/*v*) ethanol (degrees), and 184.0° is the specific optical rotation of the starch mixture. All measurements were performed using a 200 mm polarimeter tube.

The TS content of the experimental ingredients was determined using a polarimetric method. Accurately weighed samples were extracted with distilled water to transfer the soluble sugars into the liquid phase. Potassium hexacyanoferrate (II) (0.29 mol/L Carrez I) and zinc acetate (1.0 mol/L Carrez II) were subsequently added to precipitate proteins and other interfering substances, and the precipitate was removed by filtration through filter paper. The clear filtrate was transferred into a 2 dm polarimeter tube, and the optical rotation angle (α) was measured at 20 °C using a Pol-200 semiautomatic polarimeter (Bante, Sugar Land, TX, USA). The total sugar content was calculated using the specific optical rotation of sucrose at 20 °C (66.5°) as the reference value, according to the following equation:

TS (%) = (α × V × 100)/(66.5 × l × m)

where α is the observed optical rotation (°), V is the final volume of the solution (mL), l is the polarimeter tube length (dm), and m is the sample weight (g).

The apparent metabolizable energy (AME) content of the experimental ingredients was predicted from their proximate chemical composition using the predictive equation proposed by the World’s Poultry Science Association (WPSA) [18]. Accordingly, the AME values reported in this study represent composition-based predictions rather than experimentally determined metabolizable energy values. The AME content of each ingredient was calculated using the following equation:

AME(MJ/kg) = (0.1551 × CP) + (0.3431 × EE) + (0.1669 × S) + (0.1301 × TS)

### 2.3. Determination of In Vitro Crude Protein Digestibility

In vitro crude protein (CP) digestibility was determined according to a modified Stutzer method [19]. Briefly, 1.0 g of each sample was accurately weighed and incubated in an acidic solution containing 0.075 N hydrochloric acid (HCl) and pepsin from porcine gastric mucosa (Sigma-Aldrich, Burlington, MA, USA, P7000; ≥250 U/mg solid; CAS No. 9001-75-6) at 40 °C for 48 h to simulate gastric digestion. Following incubation, the digestate was filtered through a pre-weighed filter paper, and the retained residue was collected and weighed. A blank filter paper was processed under the same conditions to correct for the contribution of the filter paper to the residue weight.

The nitrogen content of the recovered residue was determined using the Kjeldahl method according to AOAC Official Method 984.13 [11]. Nitrogen was quantified by titration with 0.1 N sulfuric acid (H_2_SO_4_), and crude protein content was calculated using a nitrogen-to-protein conversion factor of 6.25. The volume of sulfuric acid consumed by the blank filter paper was subtracted from the volume measured for each sample. The crude protein content of the undigested residue was then calculated based on the corrected titration volume and residue weight.

Crude protein digestibility was calculated according to the following equation:

CP digestibility (%) = 100 − [((Undigested CP)/(Initial CP)) × 100]

where initial CP is the crude protein content of the 1.0 g sample used for the digestion assay, and undigested CP is the crude protein recovered in the filtered residue after incubation. The results were expressed as the percentage of in vitro crude protein digestibility.

### 2.4. Determination of Amino Acid Profiles

The amino acid composition of DF-BSFLM, FF-BSFLM, and SBM was determined using an Agilent LC–MS/MS system (Agilent Technologies, Santa Clara, CA, USA). Target amino acids were quantified using multiple reaction monitoring (MRM) coupled to electrospray ionization (ESI), operated in positive-ion mode. The LC–MS/MS-MRM approach was chosen to provide selective and sensitive quantification of individual amino acids in the complex, protein-rich matrices evaluated in this study. The analytical procedure was based on previously published methods [20,21].

For sample preparation, each 0.5 g sample was subjected to acid hydrolysis for 24 h at 110 °C in a screw-capped tube using 4 mL of acidic hydrolysis solution. Following hydrolysis, samples were centrifuged for 5 min at 4000 rpm using a Hettich Universal 320 air-cooled centrifuge (Andreas Hettich GmbH, Tuttlingen, Germany). An aliquot of 10 µL of the resulting supernatant was subsequently diluted 800-fold with distilled water.

Chromatographic separation was performed using a Jasem amino acid analysis column maintained at 30 °C. A 3 µL aliquot of the prepared sample was injected into the system. Separation was conducted using Jasem mobile phases A and B under gradient elution at a flow rate of 0.7 mL/min according to Nateghpour et al. [20]. The gradient program consisted of an increase in mobile phase A from 22% to 78% over 3 min, followed by a 0.5 min hold at 78% A. The mobile phase was subsequently returned to 22% A, and the column was re-equilibrated for 3 min.

Mass spectrometric detection was performed using an ESI source operated in positive-ion mode. The capillary voltage was set to 2000 V, and the drying gas temperature was maintained at 150 °C. Full-scan data were acquired over the range of 30–3000 m/z. MRM transitions for each amino acid were optimized by adjusting the fragmentation voltage and collision energy. Quantification was based on the ratio of the peak area of each amino acid to that of the corresponding internal standard, as described by Bilgin et al. [21]. Seventeen amino acids were quantified in the present study: arginine, histidine, isoleucine, leucine, lysine, methionine, phenylalanine, threonine, valine, cysteine, glycine, proline, tyrosine, alanine, aspartic acid, glutamic acid, and serine. Tryptophan was not included among the amino acids quantified in the present analytical procedure. Amino acid concentrations were expressed as g/100 g of sample.

### 2.5. Determination of Fatty Acid Profile

The fatty acid profiles of DF-BSFLM, FF-BSFLM, and SBM were determined by gas chromatography following conversion of lipids to fatty acid methyl esters (FAMEs). Analyses were performed according to the procedure described by Uguz and Sozcu [22] using an Agilent 6890N gas chromatograph (Agilent Technologies Inc., Wilmington, DE, USA) equipped with a flame ionization detector (FID).

Fatty acids were separated using an HP-88 capillary column (with 100 m × 0.25 mm internal diameter and 0.20 µm film thickness; Agilent Technologies Inc., Santa Clara, CA, USA). Then, 1 μL of the FAME solution was injected, and helium was used as the carrier gas at a flow rate of 2 mL/min. Injector and detector temperatures were maintained at 250 °C and 280 °C, respectively. The oven temperature was initially maintained at 120 °C for 1 min, was increased to 175 °C at 10 °C/min, and was held at 175 °C for 10 min. It was then increased to 210 °C at 3 °C/min, held for 5 min, then increased to 240 °C at 5 °C/min, and finally held for 5 min.

FAMEs were identified by their retention times, and quantification was performed using an internal standard (Mixture ME-100; Greyhound Chromatography and Allied Chemicals, Birkenhead, Merseyside, UK) by peak-area integration.

Individual fatty acids were expressed as percentages of the total identified fatty acids. The sums of saturated fatty acids (SFAs), monounsaturated fatty acids (MUFAs), and polyunsaturated fatty acids (PUFAs) were calculated. Total omega-3 (n-3) and omega-6 (n-6) fatty acids, as well as the UFA/SFA, PUFA/SFA, and n-6/n-3 ratios, were also calculated.

### 2.6. Determination of Total Phenolic Content

Total phenolic content (TPC) was determined using a Folin–Ciocalteu method based on the procedure described by Singleton et al. [23], with modifications. For extraction, 1.00 g of each sample was homogenized with 20 mL of 80% ethanol (1:20, *w*/*v*). Following extraction, the mixture was centrifuged at 4000× *g* for 10 min, and the supernatant was collected for analysis.

For the Folin–Ciocalteu assay, 20 µL of the sample extract was mixed with 1.58 mL of ultrapure water, 100 µL of Folin–Ciocalteu reagent, and 300 µL of 20% sodium carbonate (Na_2_CO_3_). The mixture was incubated at 40 °C for 30 min, and absorbance was measured at 765 nm using a UV–Vis spectrophotometer (Shimadzu UV-2600, Kyoto, Japan). Gallic acid was used as the reference standard to quantify TPC via a calibration curve. The results were expressed as milligrams of gallic acid equivalents per gram of sample (mg GAE/g).

### 2.7. Determination of DPPH Radical-Scavenging Activity

DPPH radical-scavenging activity was determined by an accredited analytical laboratory based on the method described by Brand-Williams et al. [24], with modifications. For extraction, 1.00 g of each sample was homogenized in 10 mL of ethanol (1:10, *w*/*v*). The extracts were subjected to ultrasonic extraction at 40 °C for 2 h and then centrifuged at 4000× *g* for 10 min at 4 °C. The supernatant was collected for the DPPH assay.

For the assay, 0.5 mL of the sample extract was mixed with 0.5 mL of a 0.5 mM DPPH solution and incubated in the dark for 30 min. Absorbance was measured using a UV–Vis spectrophotometer (Shimadzu UV-2600, Japan) at 517 nm. Trolox and ascorbic acid were used as reference antioxidants. DPPH radical-scavenging activity was quantified using a Trolox calibration curve and expressed as milligrams of Trolox equivalents per gram of sample (mg TE/g).

### 2.8. Statistical Analysis

For each ingredient, five samples were collected from different locations within a single commercial batch. Each sample was ground, homogenized, and analyzed separately. The results are presented as means and standard deviations (SDs) based on the five within-batch sampling replicates for each ingredient. Because only one commercial batch was available for each ingredient, the observed variability represents within-batch sampling variation and does not account for between-batch variability. Accordingly, the results were evaluated descriptively, without inferential statistical comparisons, and are interpreted as characterizations of the specific commercial materials examined rather than as generalizable differences among ingredient types.

## 3. Results

### 3.1. Chemical Composition and Predicted Apparent Metabolizable Energy

The nutrient composition and AME values of the three commercial protein ingredients are presented in Table 1. The DM content was similar in FF-BSFLM (97.30%) and DF-BSFLM (97.05%), whereas SBM had a lower DM content (93.26%). The CA content was highest in DF-BSFLM (11.87%), followed by FF-BSFLM (8.38%) and SBM (7.15%). CP) content was highest in DF-BSFLM (55.70%), followed by SBM (47.68%) and FF-BSFLM (39.43%). In contrast, EE content was highest in FF-BSFLM (37.09%), followed by DF-BSFLM (12.01%) and SBM (1.68%). The S content was 2.39% in DF-BSFLM, 2.05% in FF-BSFLM, and 1.32% in SBM. Total sugar content was relatively similar among the ingredients, ranging from 20.73% in DF-BSFLM to 21.40% in SBM. The predicted AME values were 21.92 MJ/kg for FF-BSFLM, 15.86 MJ/kg for DF-BSFLM, and 10.98 MJ/kg for SBM.

### 3.2. In Vitro Protein Digestibility

**Figure 1:**
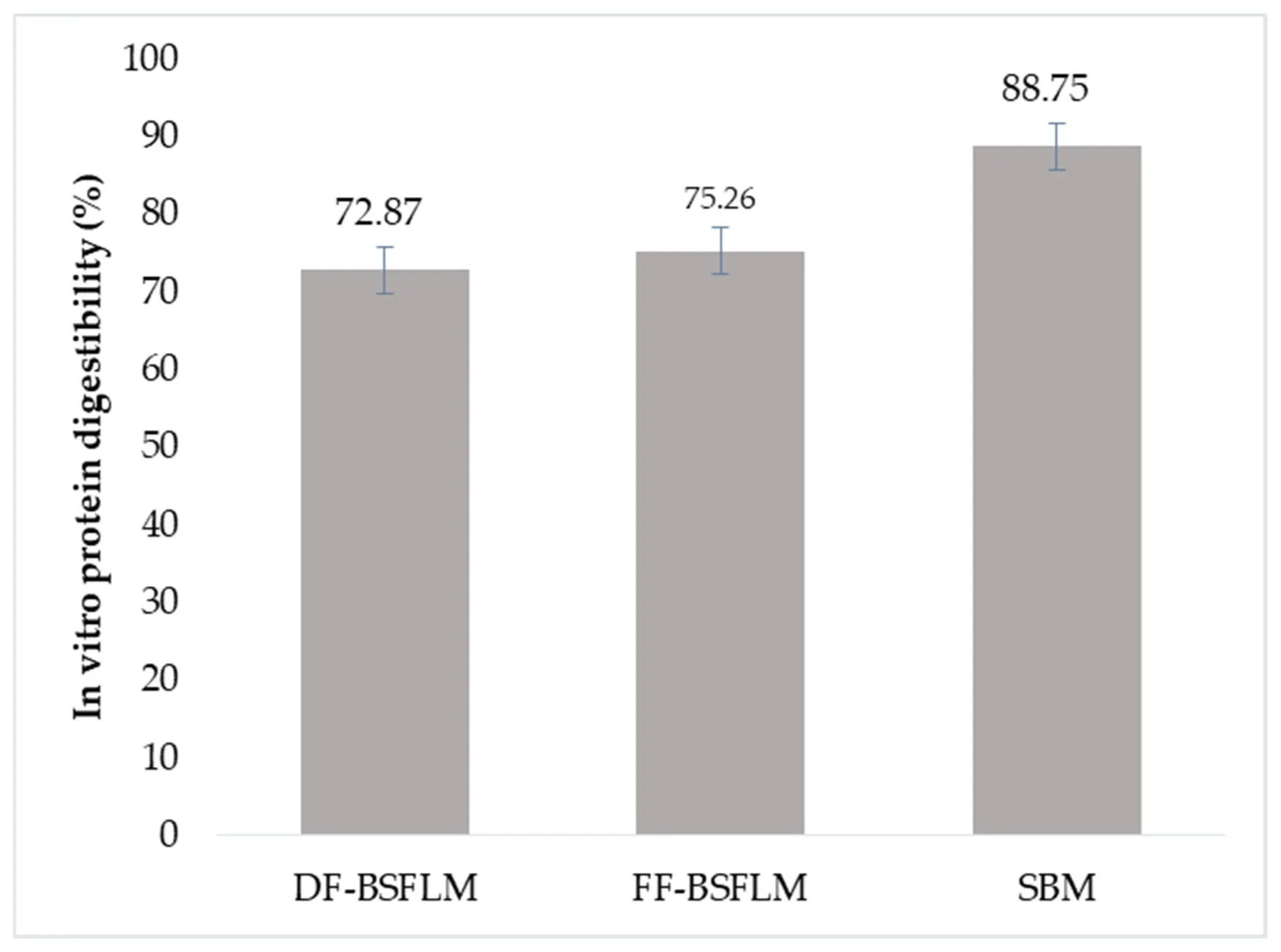
In vitro digestibility of BSFL products and SBM. DF-BSFLM: De-fatted black soldier fly larvae meal. FF-BSFLM: Full-fat black soldier fly larvae meal. SBM: Soybean meal. Values are presented as mean ± standard error (SE) of five within-batch sampling replicates per ingredient (*n* = 5).

Crude protein (CP) digestibility varied among the three commercial protein ingredients (Figure 1). SBM had the highest CP digestibility (88.75%), followed by FF-BSFLM (75.26%) and DF-BSFLM (72.87%).

### 3.3. Amino Acid Profile

The amino acid composition of the three commercial protein ingredients is presented in Table 2. Among the essential amino acids, SBM had the highest concentrations of arginine (3.10 g/100 g), isoleucine (1.10 g/100 g), leucine (3.33 g/100 g), lysine (3.00 g/100 g), and phenylalanine (2.36 g/100 g). In contrast, DF-BSFLM had higher concentrations of histidine (1.19 g/100 g), methionine (0.75 g/100 g), threonine (1.74 g/100 g), and valine (1.66 g/100 g) than FF-BSFLM. Methionine concentration was notably higher in both BSFL meals than in SBM (0.22 g/100 g).

For the conditionally essential amino acids, DF-BSFLM showed the highest concentrations of glycine (2.42 g/100 g), proline (3.04 g/100 g), and tyrosine (2.07 g/100 g), whereas SBM had the highest cysteine concentration (0.51 g/100 g). Among the non-essential amino acids, SBM had the highest concentrations of aspartic acid (5.76 g/100 g), glutamic acid (10.89 g/100 g), and serine (2.62 g/100 g), while DF-BSFLM had the highest alanine concentration (3.48 g/100 g).

The total essential amino acid (TEAA) content was 15.02, 10.62, and 17.43 g/100 g in DF-BSFLM, FF-BSFLM, and SBM, respectively. The total non-essential amino acid (TNEAA) content was 15.38, 11.18, and 21.51 g/100 g, respectively, while the total amino acid (TAA) content was 38.11, 27.57, and 45.69 g/100 g, respectively. The TEAA/TAA ratio was slightly higher in DF-BSFLM (39.42%) than in FF-BSFLM (38.58%) and SBM (38.15%). Similarly, the TEAA/NEAA ratio was highest in DF-BSFLM (97.71%), followed by FF-BSFLM (95.34%) and SBM (81.06%). These values describe the amino acid profiles of the specific commercial batches examined; no inferential statistical comparisons were performed.

### 3.4. Fatty Acid Profile

The fatty acid profiles of the three commercial protein ingredients are presented in Table 3. Among saturated fatty acids (SFAs), palmitic acid (C16:0) was the predominant fatty acid in all three ingredients, with values of 17.58% in DF-BSFLM, 20.67% in FF-BSFLM, and 15.67% in SBM. Lauric acid (C12:0) was abundant in both BSFL meals (17.18% and 18.79% in DF-BSFLM and FF-BSFLM, respectively) but was present at a very low concentration in SBM (0.03%). Stearic acid (C18:0) accounted for 6.92%, 9.54%, and 7.08% in DF-BSFLM, FF-BSFLM, and SBM, respectively. Overall, total SFA content was highest in FF-BSFLM (57.57%), followed by DF-BSFLM (49.03%), and it was lowest in SBM (26.35%).

Among monounsaturated fatty acids (MUFAs), oleic acid (C18:1 cis) was present at similar concentrations in all three ingredients, ranging from 15.86% in DF-BSFLM to 16.78% in FF-BSFLM. Palmitoleic acid (C16:1) was more abundant in the BSFL meals (3.84% and 4.25%) than in SBM (0.57%). Total MUFA content was 20.53% in DF-BSFLM, 24.44% in FF-BSFLM, and 21.28% in SBM.

The polyunsaturated fatty acid (PUFA) profiles differed among the ingredients. Alpha–linolenic acid (C18:3 n-3) was highest in SBM (12.74%), followed by FF-BSFLM (5.75%) and DF-BSFLM (1.52%). Similarly, linoleic acid (C18:2 n-6) was highest in SBM (38.59%), compared with 18.59% in FF-BSFLM and 12.40% in DF-BSFLM. Total PUFA content followed the same pattern, reaching 51.71% in SBM, 27.83% in FF-BSFLM, and 14.64% in DF-BSFLM. Consequently, total unsaturated fatty acid (UFA) content was highest in SBM (72.99%), followed by FF-BSFLM (52.27%) and DF-BSFLM (39.07%).

The n-3 fatty acid content was highest in SBM (12.82%), whereas the n-6 fatty acid content was also highest in SBM (38.89%). The n-6/n-3 ratio was 8.09 in DF-BSFLM, 2.99 in FF-BSFLM, and 2.95 in SBM. The UFA/SFA ratio was highest in SBM (2.77), followed by FF-BSFLM (0.91) and DF-BSFLM (0.68), while the PUFA/SFA ratio was 1.96 in SBM, compared with 0.26 and 0.25 in DF-BSFLM and FF-BSFLM, respectively. These results describe the fatty acid profiles of the specific commercial batches examined and are presented without inferential statistical comparisons.

### 3.5. Total Phenolic Content

**Figure 2:**
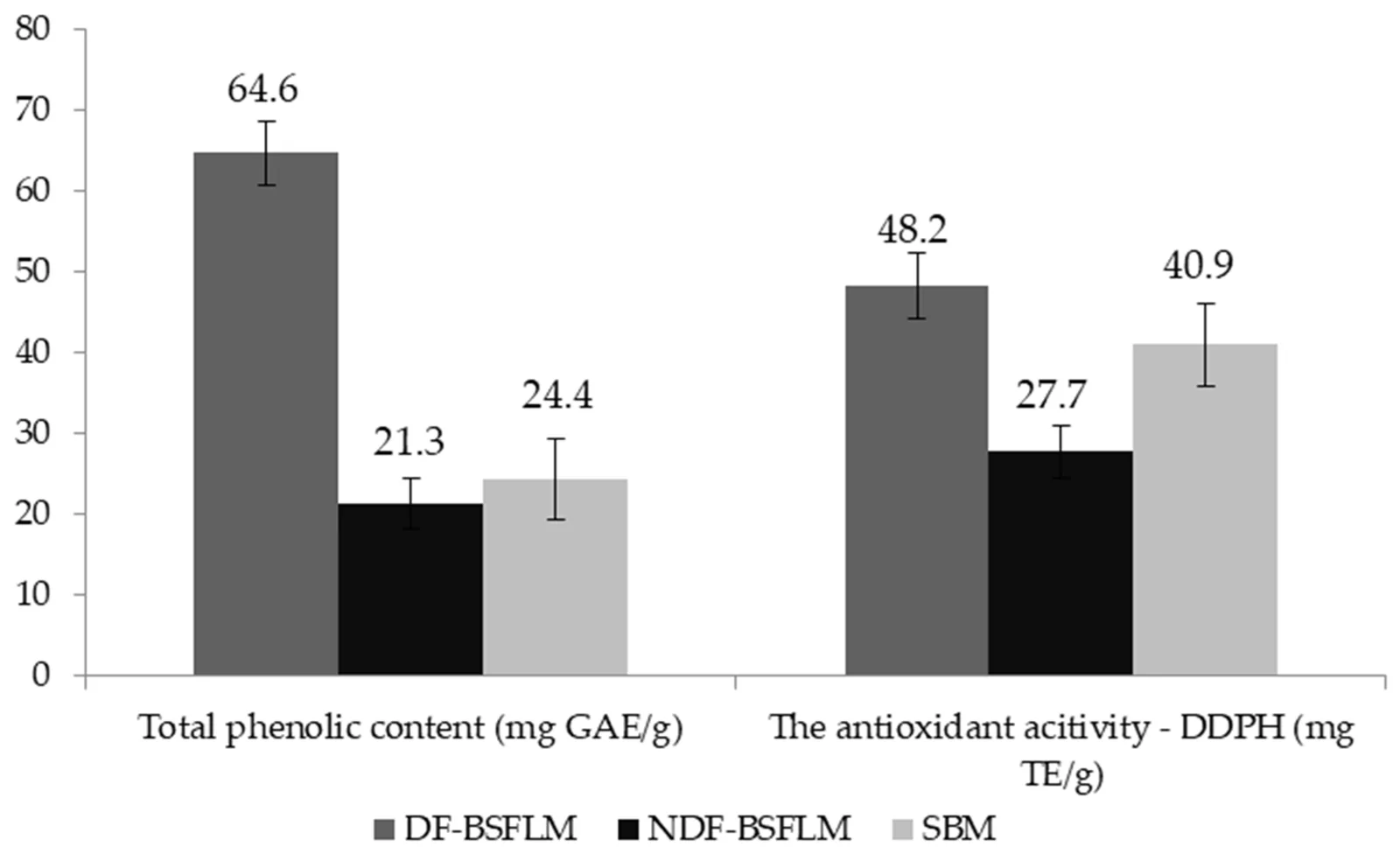
Total phenolic content and DPPH radical-scavenging activity of BSFLM products and SBM. Values are expressed on an air-dry basis and presented as mean ± standard deviation (SD) of five within-batch sampling replicates obtained from a single commercial batch of each ingredient (*n* = 5).

The TPC varied among the three commercial protein ingredients (Figure 2). DF-BSFLM had the highest TPC (64.6 mg GAE/g), followed by SBM (24.4 mg GAE/g) and FF-BSFLM (21.3 mg GAE/g).

### 3.6. Antioxidant Capacity

DPPH radical-scavenging activity varied among the three commercial protein ingredients (Figure 2). DF-BSFLM exhibited the highest activity (48.2 mg TE/g), followed by SBM (40.9 mg TE/g) and FF-BSFLM (27.7 mg TE/g).

## 4. Discussion

### 4.1. Chemical Composition and Predicted Apparent Metabolizable Energy

The present findings highlight differences in the chemical composition of the two commercial BSFLM products, FF-BSFLM and DF-BSFLM, relative to SBM. The higher dry matter (DM) content of both BSFLM products compared with SBM may be associated with the relatively low residual moisture commonly reported for processed BSFLM [25]. The higher crude ash concentration in DF-BSFLM than in FF-BSFLM may in part reflect differences in the relative proportions of non-lipid components in the two products. Similar differences in ash concentration between full-fat and defatted BSFL products have been reported previously [26]. However, ash concentration may also be influenced by larval developmental stage, rearing substrate, and processing conditions [26]. Likewise, the higher CP concentration in DF-BSFLM than in FF-BSFLM may reflect differences in the relative proportions of protein and lipid in these commercial products. Comparable differences in CP concentration between full-fat and defatted BSFLM products have been reported in previous studies [27,28]. Although both products in the present study were obtained from the same commercial supplier, they were commercially available products rather than fractions produced by defatting a common batch. Therefore, the observed CP difference should be interpreted as a difference between the specific commercial products evaluated, rather than as a direct causal effect of defatting. Comparisons between insect meals and SBM should also account for the potential overestimation of true protein content in insect meals by conventional nitrogen-based methods, because non-protein nitrogen may contribute to the measured CP value [29].

The EE content was substantially higher in FF-BSFLM (37.09%) than in DF-BSFLM (12.01%) and SBM (1.68%), highlighting differences in lipid content among the three feed ingredients. BSF larvae can accumulate considerable amounts of lipids, and their lipid content may vary with rearing substrate, developmental stage, and processing conditions [30]. These compositional differences are relevant when considering the potential nutritional applications of BSFLM products. In contrast, SBM generally contains less residual lipid because oil is removed during soybean processing. The low EE content of SBM observed in this study is consistent with its characterization as a protein-rich, relatively low-fat feed ingredient.

Starch concentrations were numerically low in all three ingredients, ranging from 1.32% in SBM to 2.39% in DF-BSFLM. These values are consistent with the general composition of insect meals, which are characterized primarily by protein and lipid rather than starch [31]. The differences in starch concentration among the evaluated products may reflect variation in their carbohydrate fractions and processing histories; however, the present data do not establish the specific factors responsible for these differences. Total sugar concentrations were relatively similar across the three ingredients, ranging from 20.73% in DF-BSFLM to 21.40% in SBM. Despite their different biological origins, the products therefore showed comparable total sugar concentrations. Nevertheless, similar total sugar values do not necessarily indicate equivalent carbohydrate composition or availability, and further characterization would be required to establish the nutritional significance of these sugars [31].

The predicted AME values were highest for FF-BSFLM (21.92 MJ/kg), followed by DF-BSFLM (15.86 MJ/kg) and SBM (10.98 MJ/kg). The higher predicted AME of FF-BSFLM is consistent with its greater EE concentration, as lipid contributes substantially to the energy value estimated by the applied chemical composition-based equation. Differences in the nutrient composition and processing characteristics of BSFLM products may consequently contribute to variation in their predicted energy values [29,30,31]. However, because AME was estimated from chemical composition rather than determined experimentally in feeding trials, these values should be interpreted as predictions and not as directly measured metabolizable energy. The higher predicted AME of FF-BSFLM primarily reflects the greater lipid concentration of this product within the applied equation, whereas the lower predicted values for DF-BSFLM and SBM are consistent with their lower EE concentrations. Overall, this comparison integrates the energy-related characteristics of the specific commercial FF-BSFLM, DF-BSFLM, and SBM products examined, without attributing the observed differences solely to defatting or generalizing them to all products within these ingredient categories.

### 4.2. In Vitro Protein Digestibility

In vitro CP digestibility results indicated that the higher CP concentration in DF-BSFLM was not accompanied by greater protein digestibility. CP digestibility was 72.87% for DF-BSFLM and 75.26% for FF-BSFLM, whereas SBM showed a higher value of 88.75%. Previous studies have reported differences in the protein digestibility of full-fat and defatted BSFL products, with some reporting greater in vitro digestibility for defatted BSFL protein than for dried, non-defatted larvae [32]. However, the magnitude and direction of these differences may depend on processing conditions, product characteristics, and the experimental system used to assess digestibility. Because the present study evaluated commercially available FF- and DF-BSFLM products rather than material obtained by defatting a common batch, the observed differences should be interpreted as product-level differences rather than direct effects of defatting. The lower in vitro CP digestibility of both BSFLM products compared with SBM may reflect differences in protein characteristics and ingredient matrices rather than CP concentration alone.

Differences in processing and the resulting protein matrix may partly explain the numerical difference in digestibility between DF-BSFLM and FF-BSFLM; however, the underlying mechanisms cannot be established from the present data. Structural components such as chitin may influence protein accessibility and digestion in insect-derived ingredients. Recent in vitro evidence indicates that chitin-rich fractions of BSFL exhibit lower protein solubilization and hydrolysis than protein-rich fractions [33]. Nevertheless, chitin content and protein structural characteristics were not directly determined in the present study, and these factors should therefore be considered possible explanations rather than established causes.

The results highlight the importance of evaluating CP concentration alongside protein digestibility when assessing BSFLM as a protein ingredient. A higher CP concentration does not necessarily indicate greater protein digestibility or availability. Previous studies in broilers have likewise emphasized the importance of considering nutrient and amino acid digestibility in addition to proximate composition when evaluating BSFLM [32,33,34]. However, the in vitro digestibility values obtained in the present study should be interpreted within the context of the assay conditions and should not be taken as direct evidence of inferior overall protein quality or lower nutritional value in poultry. From a poultry nutrition perspective, further assessment of amino acid availability, particularly for lysine, methionine, and threonine, would help clarify the nutritional characteristics of these products. In vivo protein utilization studies and standardized ileal amino acid digestibility measurements would provide a more comprehensive assessment of the practical feeding value of commercially available FF- and DF-BSFLM products.

### 4.3. Amino Acid Profile

The differences in amino acid concentrations among the three ingredients indicate that BSFLM and SBM have distinct amino acid profiles. Although SBM had higher concentrations of several essential amino acids, DF-BSFLM showed relatively high concentrations of certain amino acids, highlighting differences between these specific insect- and plant-derived protein ingredients.

The particularly high methionine concentration in DF-BSFLM is noteworthy, as it was more than three times that of SBM in the present study. This finding differs from the commonly reported pattern in which methionine is among the relatively less abundant essential amino acids in BSFLM [2,35]. Variation in larval nutrition, substrate composition, rearing conditions, processing history, and analytical procedures may contribute to such differences [34,36]. This result highlights the variability that may occur among commercially available BSFL products, which should be considered when evaluating their composition and formulating poultry diets. However, differences in amino acid concentrations reflect compositional variation and do not, by themselves, establish differences in protein quality, particularly because amino acid digestibility and metabolic availability were not determined in the present study.

From a poultry nutrition perspective, the concentrations of methionine, lysine, and threonine in DF-BSFLM may be relevant because these amino acids require particular attention during poultry diet formulation [37]. Nevertheless, their nutritional contribution depends not only on their concentrations but also on their digestibility and availability. Previous studies have demonstrated that BSFLM can provide appreciable amounts of digestible essential amino acids to poultry, supporting its potential as an alternative protein ingredient [38].

Although SBM had the highest TAA concentration, DF-BSFLM had the highest TEAA/TAA and TEAA/TNEAA ratios among the three ingredients. These ratios indicate that essential amino acids represented a relatively larger proportion of the measured amino acid pool in the BSFLM products than in SBM. However, they should be interpreted as indicators of amino acid distribution rather than direct measures of protein quality. The nutritional contribution of each ingredient depends on the digestibility and availability of individual amino acids. Accordingly, standardized ileal amino acid digestibility measurements are needed to determine whether the distinct amino acid profiles of these commercial products translate into meaningful differences in essential amino acid availability for poultry [34,35].

### 4.4. Fatty Acid Profile

The distinct fatty acid profiles observed among the tested ingredients highlight an important nutritional difference between BSFLM and SBM. The predominance of SFAs, particularly lauric acid, in BSFLM is consistent with the characteristic lipid composition of BSF. Lauric, myristic, palmitic, and stearic acids are among the major saturated fatty acids commonly reported in BSFL [38]. Their relative abundance can vary depending on larval diet, rearing substrate, production conditions, and processing history [38,39].

The higher SFA concentration observed in FF-BSFLM coincided with its higher overall lipid content, whereas SBM had a PUFA-rich fatty acid profile. In particular, linoleic acid (38.59%) and alpha–linolenic acid (12.74%) accounted for a substantial proportion of the total PUFA concentration in SBM (51.71%). Conventional soybean-derived ingredients are generally characterized by a PUFA-rich lipid profile, particularly with respect to linoleic acid, although the fatty acid composition may vary according to soybean genotype and processing conditions [39]. These differences suggest that replacing SBM with BSFLM may alter not only the protein supply but also the fatty acid contribution of the diet.

The EPA concentration observed in FF-BSFLM is also noteworthy. Fatty acid composition in BSFL can be influenced by the composition of the rearing substrate, and the inclusion of marine-derived materials has been reported to increase long-chain n-3 fatty acids [30,36]. The EPA profile of the tested FF-BSFLM product may therefore reflect its specific rearing or production conditions. However, this potential explanation cannot be verified from the present data because the composition of the rearing substrate and the production history of the commercial product were not evaluated.

The higher n-6/n-3 ratio observed in DF-BSFLM was associated with its comparatively low n-3 PUFA concentration. Nevertheless, this ratio should not be interpreted independently of the absolute concentrations and individual fatty acid composition. The nutritional significance of a lipid source depends on the amounts and biological characteristics of individual fatty acids rather than on the n-6/n-3 ratio alone.

The tested FF-BSFLM and DF-BSFLM products differed in both fatty acid profiles and total lipid concentrations. Previous research has indicated that harvesting, drying, and processing procedures may contribute to variation in the fatty acid composition of BSFL, particularly in the proportions of lauric, palmitic, and myristic acids [38]. Although both BSFLM products evaluated in the present study were obtained from the same commercial supplier, they represented commercially available products rather than experimentally generated FF and DF fractions derived from a common batch under controlled processing conditions. Consequently, the observed differences cannot be attributed exclusively to defatting. Variations in raw materials, larval diet, rearing conditions, processing history, and other product-specific characteristics may also have contributed to the distinct fatty acid profiles. Accordingly, the findings should be interpreted as a descriptive, product-level characterization of the tested commercial BSFLM products in comparison with SBM, rather than as an experimental assessment of the causal effects of defatting.

### 4.5. Total Phenolic Content

The higher TPC observed in DF-BSFLM indicates a greater Folin–Ciocalteu-reactive content than that of the other ingredients evaluated in the present study. Differences in TPC among the tested ingredients may reflect variations in the chemical matrix, processing history, production conditions, or other product-specific characteristics that can affect the concentration and extractability of phenolic and other reducing compounds. Recent evidence indicates that processing conditions can influence the nutritional, functional, and bioactive characteristics of BSF products, although the nature and extent of these effects may depend on the specific processing conditions and product characteristics [10].

The TPC of DF-BSFLM was higher than that of SBM, whereas FF-BSFLM showed a TPC comparable to that of SBM. These observations indicate that the commercially available BSFLM products evaluated in the present study differed in their Folin–Ciocalteu-reactive characteristics. However, the higher TPC observed in DF-BSFLM should not be interpreted as direct evidence that defatting increased phenolic content or antioxidant potential. This distinction is important because the present study compared commercially available FF- and DF-BSFLM products rather than experimentally generated fractions derived from a common batch under controlled processing conditions. In addition, the Folin–Ciocalteu assay is not specific to phenolic compounds and may respond to other reducing substances present in the sample matrix. Therefore, the higher TPC observed in the commercial DF-BSFLM product may reflect differences in production conditions, rearing substrate, processing history, or other product-specific characteristics [40]. Because these factors were not controlled or independently evaluated in the present study, their individual contributions to the observed differences cannot be established.

The higher TPC observed in DF-BSFLM represents a distinct in vitro characteristic of the tested product and contributes to the integrated functional characterization of the commercially available BSFLM products. Phenolic compounds and other reducing constituents may contribute to the chemical and functional properties of feed ingredients; however, a higher TPC measured in vitro does not necessarily indicate a physiological antioxidant effect in poultry. Therefore, the biological significance of the observed TPC profile requires further investigation using more specific chemical analyses and controlled in vivo studies.

### 4.6. Antioxidant Capacity

The DPPH radical-scavenging activity differed among the ingredients evaluated in the present study. DF-BSFLM showed a DPPH activity of 48.2 mg TE/g, compared with 40.9 mg TE/g for SBM and 27.7 mg TE/g for FF-BSFLM. Previous research has shown that the radical-scavenging characteristics of *H. illucens* products can vary with production and processing conditions, including extraction conditions [10,40]. Differences in the chemical matrix of commercially available BSFLM products may also influence the concentration and extractability of compounds capable of reacting with DPPH radicals. Therefore, the DPPH activity observed in DF-BSFLM should be considered a characteristic of the specific commercial product evaluated rather than direct evidence of an effect attributable to defatting. Although the DPPH activity represents a distinct functional characteristic of DF-BSFLM under the present assay conditions, it should not be interpreted as direct evidence of superior nutritional or physiological effects in poultry.

The higher DPPH activity observed in DF-BSFLM coincided with its higher TPC (64.6 mg GAE/g) relative to the other ingredients. Nevertheless, this relationship should be interpreted cautiously. The DPPH assay provides an in vitro estimate of radical-scavenging activity and does not constitute direct evidence of physiological antioxidant effects in poultry. Moreover, DPPH activity cannot be attributed exclusively to phenolic compounds, as peptides and other bioactive constituents may also contribute to the measured response. The differences in DPPH activity and TPC profiles among the tested ingredients indicate that radical-scavenging activity cannot be explained solely by total Folin–Ciocalteu-reactive content.

Previous research has shown that antioxidant-related characteristics of BSFL can vary according to the rearing substrate [10]. Therefore, production conditions, rearing substrates, harvesting practices, processing histories, and extraction conditions should be considered when interpreting differences in the functional properties of commercially available BSFLM products. Because these factors were not independently controlled or evaluated in the present study, their individual contributions to the observed DPPH activity profiles cannot be established.

From a poultry nutrition perspective, the DPPH activity of DF-BSFLM relative to SBM represents a distinct in vitro characteristic of the specific commercial product evaluated in the present study. However, this observation should not be interpreted as evidence of a direct effect of defatting because the production, rearing substrate, processing, and extraction conditions associated with the commercial products were not independently controlled. Moreover, the extent to which differences in in vitro radical-scavenging activity may be associated with oxidative status, intestinal health, nutrient utilization, or productive performance in poultry remains uncertain. Therefore, the functional characteristics of commercially available BSFLM products should be considered alongside nutrient digestibility and amino acid availability rather than evaluated solely on the basis of DPPH activity. Controlled in vivo studies are needed to determine whether differences in in vitro radical-scavenging activity among BSFLM products are associated with measurable physiological responses or productive outcomes in poultry.

An important limitation of the present study is that each ingredient was represented by a single commercial batch, with five analytical replicates obtained from the same homogenized material. Consequently, the observed differences characterize the specific commercial batches evaluated and should not be interpreted as estimates of batch-to-batch variability or generalized to all commercially available DF-BSFLM, FF-BSFLM, or SBM products. Studies involving multiple independent commercial batches are needed to assess product- and batch-level variability and support broader generalization.

## 5. Conclusions

The present study provided an integrated comparative characterization of commercially available defatted black soldier fly larvae meal (DF-BSFLM), full-fat black soldier fly larvae meal (FF-BSFLM), and soybean meal (SBM) based on nutritional composition, predicted energy value, in vitro protein digestibility, amino acid and fatty acid profiles, and in vitro functional characteristics. DF-BSFLM had a higher crude protein concentration, whereas FF-BSFLM had a higher ether extract concentration and predicted apparent metabolizable energy. The predicted AME values were 15.86, 21.92, and 10.98 MJ/kg DM for DF-BSFLM, FF-BSFLM, and SBM, respectively. In vitro crude protein digestibility values were 72.87%, 75.26%, and 88.75%, respectively. SBM had higher concentrations of several essential amino acids and polyunsaturated fatty acids, whereas the BSFLM products were characterized by higher proportions of saturated fatty acids, particularly lauric acid. DF-BSFLM also showed higher total Folin–Ciocalteu-reactive content and in vitro DPPH radical-scavenging activity than the other tested ingredients.

Because the FF- and DF-BSFLM products were commercially available materials rather than experimentally generated fractions obtained by defatting a common batch under controlled conditions, the observed differences cannot be attributed exclusively to the defatting process. Furthermore, each ingredient was represented by a single commercial batch, with five analytical replicates obtained from the same homogenized material. Therefore, the findings characterize the specific batches evaluated and do not establish batch-to-batch variability or support generalization to all commercially available products. The predicted AME values should be interpreted as composition-based estimates rather than experimentally determined energy values, while the DPPH results represent in vitro radical-scavenging activity rather than evidence of physiological antioxidant effects. Overall, this integrated product-level characterization provides comparative information on the nutritional composition, digestibility, fatty acid profiles, and in vitro functional characteristics of the tested BSFLM products relative to SBM. Further studies involving multiple independent commercial batches and controlled in vivo trials are needed to assess product variability and determine nutrient bioavailability, energy utilization, standardized ileal amino acid digestibility, and physiological responses in poultry.

## Figures and Tables

**Table 1:** Chemical composition of black soldier fly larvae meal (defatted and full-fat) and soybean meal.

Nutrient Composition (%)	DF-BSFLM	FF-BSFLM	SBM
Dry matter	97.05 ± 0.02	97.30 ± 0.01	93.26 ± 0.02
Crude ash	11.87 ± 0.02	8.38 ± 0.01	7.15 ± 0.05
Crude protein	55.70 ± 0.20	39.43 ± 0.13	47.68 ± 0.19
Ether extract	12.01 ± 0.25	37.09 ± 0.17	1.68 ± 0.25
Starch	2.39 ± 0 08	2.05 ± 0.13	1.32 ± 0.06
Total sugar	20.73 ± 0.04	21.05 ± 0.04	21.40 ± 0.08
AME * (MJ/kg)	15.86 ± 0.21	21.92 ± 0.11	10.98 ± 0.12

DF-BSFLM: De-fatted black soldier fly larvae meal. FF-BSFLM: Full-fat black soldier fly larvae meal. SBM: Soybean meal. Values are expressed on an air-dry basis and presented as mean ± standard deviation (SD) of five within-batch sampling replicates obtained from a single commercial batch of each ingredient (*n* = 5). * Example calculation of AME (MJ/kg): AME (MJ/kg) = (0.1551 × CP) + (0.3431 × EE) + (0.1669 × starch) + (0.1301 × total sugar); For DF-BSFLM AME (MJ/kg) = (0.1551 × 55.70) + (0.3431 × 12.01) + (0.1669 × 2.39) + (0.1301 × 20.73) = 15.86 MJ/kg.

**Table 2:** Amino acid composition of black soldier fly larvae meal (defatted and full-fat) and soybean meal.

Amino Acids (g/100 g)	DF-BSFLM	FF-BSFLM	SBM
Essential amino acids			
Arginine (Arg)	2.06 ± 0.03	1.50 ± 0.07	3.10 ± 0.21
Histidine (His)	1.19 ± 0.01	0.94 ± 0.06	1.19 ± 0.07
Isoleucine (Ile)	0.80 ± 0.01	0.49 ± 0.03	1.10 ± 0.09
Leucine (Leu)	2.64 ± 0.01	1.77 ± 0.04	3.33 ± 0.02
Lysine (Lys)	2.54 ± 0.01	1.87 ± 0.04	3.00 ± 0.02
Methionine (Met)	0.75 ± 0.01	0.63 ± 0.03	0.22 ± 0.02
Phenylalanine (Phe)	1.64 ± 0.02	1.19 ± 0.06	2.36 ± 0.04
Threonine (Thr)	1.74 ± 0.02	1.19 ± 0.8	1.77 ± 0.09
Valine (Val)	1.66 ± 0.08	1.03 ± 0.10	1.37 ± 0.25
Conditionally essential amino acids			
Cysteine (Cys)	0.18 ± 0.001	0.14 ± 0.004	0.51 ± 0.03
Glycine (Gly)	2.42 ± 0.15	1.84 ± 0.17	2.27 ± 0.16
Proline (Pro)	3.04 ± 0.02	2.17 ± 0.01	2.85 ± 0.04
Tyrosine (Tyr)	2.07 ± 0.06	1.61 ± 0.05	1.12 ± 0.08
Non-essential amino acids			
Alanine (Ala)	3.48 ± 0.02	2.13 ± 0.06	2.23 ± 0.11
Aspartic acid (Asp)	3.98 ± 0.04	3.18 ± 0.07	5.76 ± 0.06
Glutamic acid (Glu)	5.95 ± 0.31	4.46 ± 0.22	10.89 ± 0.58
Serine (Ser)	1.96 ± 0.03	1.42 ± 0.06	2.62 ± 0.17
TEAA	15.02 ± 0.03	10.62 ± 0.06	17.43 ± 0.32
TNEAA	15.38 ± 0.04	11.18 ± 0.06	21.51 ± 0.62
TAA	38.11 ± 0.09	27.57 ± 0.04	45.69 ± 0.33
TEAA/TAA (%)	39.42 ± 0.19	38.58 ± 0.18	38.15 ± 0.35
TEAA/NEAA (%)	97.71 ± 0.53	95.34 ± 0.26	81.06 ± 0.34

DF-BSFLM: De-fatted black soldier fly larvae meal. FF-BSFLM: Full-fat black soldier fly larvae meal. SBM: Soybean meal. Values are expressed on an air-dry basis and presented as mean ± standard deviation (SD) of five within-batch sampling replicates obtained from a single commercial batch of each ingredient (*n* = 5). TEAA: total essential amino acids; TNEAA: total non-essential amino acids; TAA: total amino acids. The reported amino acid concentrations should be interpreted in light of acid hydrolysis, since the recovery of individual amino acids may be affected by the hydrolysis conditions.

**Table 3:** Fatty acid profile of black soldier fly larvae meal (defatted and full-fat) and soybean meal.

Fatty Acids	DF-BSFLM	FF-BSFLM	SBM
Saturated fatty acids (SFAs, %)			
Lauric acid (C12:0)	17.18 ± 0.07	18.79 ± 0.24	0.03 ± 0.01
Myristic acid (C14:0)	5.93 ± 0.02	6.60 ± 0.07	0.60 ± 0.03
Pentadecanoic acid (C15:0)	0.28 ± 0.04	0.31 ± 0.03	0.14 ± 0.01
Palmitic acid (C16:0)	17.58 ± 0.04	20.67 ± 0.08	15.67 ± 0.05
Heptadecanoic acid (C17:0)	0.37 ± 0.04	0.88 ± 0.22	0.01 ± 0.001
Stearic acid (C18:0)	6.92 ± 0.09	9.54 ± 0.16	7.08 ± 0.04
Arachidic acid (C20:0)	0.24 ± 0.01	0.61 ± 0.04	1.17 ± 0.05
Behenic acid (C22:0)	0.00	0.00	1.19 ± 0.07
Lignoceric acid (C24:0)	0.02 ± 0.001	0.17 ± 0.001	0.45 ± 0.05
Mono-unsaturated fatty acids (MUFAs, %)			
Palmitoleic acid (C16:1)	3.84 ± 0.02	4.25 ± 0.15	0.57 ± 0.01
Oleic acid (C18:1 cis)	15.86 ± 0.06	16.78 ± 0.24	16.67 ± 0.05
Elaidic acid (C18:1 trans)	0.70 ± 0.01	1.15 ± 0.07	2.64 ± 0.01
Eicosenoic acid (C20:1 cis-11)	0.08 ± 0.05	1.15 ± 0.05	1.00 ± 0.02
Nervonic acid (C24:1 cis-15)	0.04 ± 0.01	1.11 ± 0.06	0.40 ± 0.04
Poly-unsaturated fatty acids (PUFAs), Omega-3 (n-3, %)			
Eicosapentaenoic acid (C20:5 cis-5,8,11,14,17)	0.09 ± 0.02	1.23 ± 0.02	0.08 ± 0.01
Alpha–linolenic acid (C18:3 cis-9,12,15)	1.52 ± 0.09	5.75 ± 0.04	12.74 ± 0.08
Poly-unsaturated fatty acids (PUFAs), Omega-6 (n-6, %)			
Linoleic acid (C18:2 cis-9,12)	12.40 ± 0.30	18.59 ± 0.25	38.59 ± 0.23
Gamma–linolenic acid (C18:3 cis-6,9,12)	0.16 ± 0.004	0.40 ± 0.008	0.09 ± 0.001
Arachidonic acid (C20: cis5,8,11,14)	0.07 ± 0.11	1.49 ± 0.14	0.03 ± 0.11
Eicosadienoic acid (C20:2 cis-11,14)	0.14 ± 0.04	0.22 ± 0.07	0.17 ± 0.02
Eicosatrienoic acid (C20:3 cis-8,11,14)	0.01 ± 0.004	0.15 ± 0.004	0.01 ± 0.007
SFA	49.03 ± 0.29	57.57 ± 0.54	26.35 ± 0.27
UFA	39.07 ± 0.38	52.27 ± 0.27	72.99 ± 0.33
MUFA	20.53 ± 0.12	24.44 ± 0.08	21.28 ± 0.04
PUFA	14.64 ± 0.43	27.83 ± 0.15	51.71 ± 0.22
n-3	1.61 ± 0.08	6.98 ± 0.04	12.82 ± 0.19
n-6	13.02 ± 0.24	20.85 ± 0.15	38.89 ± 0.22
UFA/SFA	0.68 ± 0.002	0.91 ± 0.006	2.77 ± 0.001
PUFA/SFA	0.26 ± 0.005	0.25 ± 0.003	1.96 ± 0.001
n-6/n-3	8.09 ± 0.26	2.99 ± 0.30	2.95 ± 0.33

DF-BSFLM: De-fatted black soldier fly larvae meal; FF-BSFLM: Full-fat black soldier fly larvae meal; SBM: Soybean meal. Values are expressed on an air-dry basis and presented as mean ± standard deviation (SD) of five within-batch sampling replicates obtained from a single commercial batch of each ingredient (*n* = 5).

## Data Availability

All datasets collected and analyzed during the current study are available from the corresponding author on fair request.

## References

[1] Thornton P., Gurney-Smith H., Wollenberg E. (2023). Alternative sources of protein for food and feed. Curr. Opin. Environ. Sustain..

[2] Hancz C., Sultana S., Nagy Z., Biró J. (2024). The role of insects in sustainable animal feed production for environmentally friendly agriculture: A review. Animals.

[3] Ibáñez M.A., de Blas C., Cámara L., Mateos G.G. (2020). Chemical composition, protein quality and nutritive value of commercial soybean meals produced from beans from different countries: A meta-analytical study. Anim. Feed. Sci. Technol..

[4] Deng Z., Kim S.W. (2024). Opportunities and challenges of soy proteins with different processing applications. Antioxidants.

[5] Siddiqui S.A., Gadge A.S., Hasan M., Rahayu T., Povetkin S.N., Fernando I., Castro-Muñoz R. (2024). Future opportunities for products derived from black soldier fly (BSF) treatment as animal feed and fertilizer—A systematic review. Environ. Dev. Sustain..

[6] Hosseindoust A., Ha S.H., Mun J.Y., Kim J.S. (2024). A meta-analysis to evaluate the effects of substrate sources on the nutritional performance of black soldier fly larvae: Implications for sustainable poultry feed. Poult. Sci..

[7] Silva B.C.R., Lehnen C.R., Marcato S.M. (2024). Black soldier fly (*Hermetia illucens*) as a protein ingredient in poultry feed. World’s Poult. Sci. J..

[8] Chobanova S., Karkelanov N., Mansbridge S.C., Whiting I.M., Tukša M., Rose S.P., Pirgozliev V.R. (2024). Metabolizable energy value of fat and meals obtained from black soldier fly larvae (*Hermetia illucens*) for broiler chickens. Poultry.

[9] Xin Y., Xu M., Chen L., Wang G., Lu W., Liu Z., Shang R., Li Y., Wang Z., Sun H. (2024). Effects of different defatting methods of black soldier fly (*Hermetia illucens*) larvae meal on the metabolic energy and nutrient digestibility in young laying hens. Animals.

[10] Marasca N.S., Araújo A.C.S., Noda K.S., Farias B.S., Brizio A.P.D.R., Fernandes S.S., Martins V.G. (2025). Effect of defatting method on the nutritional, functional, and bioactive properties of black soldier fly (*Hermetia illucens*) larvae. Insects.

[11] Barragán-Fonseca K.B., Dicke M., van Loon J.J.A. (2017). Nutritional value of the black soldier fly (*Hermetia illucens* L.)and its suitability as animal feed—A review. J. Insects Food Feed..

[12] Wang Y.-S., Shelomi M. (2017). Review of black soldier fly (*Hermetia illucens*) as animal feed and human food. Foods.

[13] Zozo B., Wicht M.M., Mshayisa V.V., van Wyk J. (2022). The nutritional quality and structural analysis of black soldier fly larvae flour before and after defatting. Insects.

[14] Schiavone A., De Marco M., Martínez S., Dabbou S., Renna M., Madrid J., Hernández F., Rotolo L., Costa P., Gai F. (2017). Nutritional value of a partially defatted and a highly defatted black soldier fly larvae (*Hermetia illucens L.*) meal for broiler chickens: Apparent nutrient digestibility, apparent metabolizable energy and apparent ileal amino acid digestibility. J. Anim. Sci. Biotechnol..

[15] Rodríguez-González E., da Cunha-Borges V., Cantero-Bahillo E., Fornari T., García-Risco M.R., Martín D. (2025). Black soldier fly (*Hermetia illucens*) larvae accumulate bioactive compounds that modulate antioxidant activity when reared with bioactive agrifood by-products. Food Res. Int..

[16] Association of Official Analytical Chemists (2006). Official Methods of Analysis of AOAC International.

[17] European Commission (2024). Commission Implementing Regulation(EU)2024/771 of 29 February 2024 amending Regulation(EC)No 152/2009 laying down the methods of sampling and analysis for the official control of feed. Off. J. Eur. Union.

[18] World’s Poultry Science Association(WPSA) (1989). European Table of Energy Values for Poultry Feedstuffs.

[19] Stutzer A. (1880). Die Einwirkung des sauren Magensaftes auf die stickstoffhaltigen Bestandtheile der Mohnkuchen. J. Landwirtsch..

[20] Nateghpour B., Kavoosi G., Mirakhorli N. (2021). Amino acid profile of the peel of three citrus species and its effect on the combination of amino acids and fatty acids *Chlorella vulgaris*. J. Food Compos. Anal..

[21] Bilgin Ö., Çarlı U., Erdoğan S., Maviş M.E., Göksu-Gürsu G., Yılmaz M. (2019). Determination of amino acids composition in different tissues of whiting, *Merlangus merlangus* euxinus (Nordmann, 1840) from the Black Sea, Turkey. Alınteri J. Agric. Sci..

[22] Uguz S., Sozcu A. (2023). Nutritional value of microalgae and cyanobacteria produced with batch and continuous cultivation: Potential use as feed material in poultry nutrition. Animals.

[23] Singleton V.L., Orthofer R., Lamuela-Raventós R.M. (1999). Analysis of total phenols and other oxidation substrates and antioxidants by means of Folin–Ciocalteu reagent. Methods Enzymol..

[24] Brand-Williams W., Cuvelier M.E., Berset C. (1995). Use of a free radical method to evaluate antioxidant activity. LWT Food Sci. Technol..

[25] Facey H., Kithama M., Mohammadigheisar M., Huber L.-A., Shoveller A.K., Kiarie E.G. (2023). Complete replacement of soybean meal with black soldier fly larvae meal in feeding program for broiler chickens from placement through to 49 days of age reduced growth performance and altered organ morphology. Poult. Sci..

[26] Meneguz M., Schiavone A., Gai F., Dama A., Lussiana C., Renna M., Gasco L., Biasato I. (2022). Biowaste and by-products as rearing substrates for black soldier fly (*Hermetia illucens*) larvae: Effects on larval body composition and performance. PLoS ONE.

[27] Makkar H.P.S., Tran G., Heuzé V., Ankers P. (2014). State-of-the-art on use of insects as animal feed. Anim. Feed. Sci. Technol..

[28] Hopkins I., Newman L.P., Gill H., Danaher J. (2021). The influence of food waste rearing substrates on black soldier fly protein composition: A systematic review. Insects.

[29] Smets R., Claes J., Van Der Borght M. (2021). On the nitrogen content and a robust nitrogen-to-protein conversion factor of black soldier fly larvae (*Hermetia illucens*). Anal. Bioanal. Chem..

[30] Hurtado-Ribeira R., Villanueva-Bermejo D., García-Risco M.R., Hernández M.D., Sánchez-Muros M.J., Fornari T., Vázquez L., Martin D. (2023). Evaluation of the interrelated effects of slaughtering, drying, and defatting methods on the composition and properties of black soldier fly (*Hermetia illucens*) larvae fat. Curr. Res. Food Sci..

[31] Meneguz M., Schiavone A., Gai F., Dama A., Lussiana C., Renna M., Gasco L. (2018). Effect of rearing substrate on growth performance, waste reduction efficiency and chemical composition of black soldier fly (*Hermetia illucens*) larvae. J. Sci. Food Agric..

[32] Traksele L., Speiciene V., Smicius R., Alencikiene G., Salaseviciene A., Garmiene G., Zigmantaite V., Grigaleviciute R., Kucinskas A. (2021). Investigation of in vitro and in vivo digestibility of black soldier fly (*Hermetia illucens* L.) larvae protein. J. Funct. Foods.

[33] Bonomini M.G., Prandi B., Caligiani A. (2024). Black soldier fly (*Hermetia illucens* L.) whole and fractionated larvae: In vitro protein digestibility and effect of lipid and chitin removal. Food Res. Int..

[34] De Marco M., Martínez S., Hernandez F., Madrid J., Gai F., Rotolo L., Belforti M., Bergero D., Schiavone A. (2015). Nutritional value of two insect meals (*Tenebrio molitor* and *Hermetia illucens* larvae meal) for broiler chickens: Apparent nutrient digestibility, apparent metabolizable energy and apparent ileal amino acid digestibility. Anim. Feed. Sci. Technol..

[35] Lemme A., Klüber P. (2024). Rethinking amino acid nutrition of black soldier fly larvae(Hermetia illucens)based on insights from an amino acid reduction trial. Insects.

[36] Kidd M.T., Maynard C.W., Mullenix G.J. (2021). Progress of amino acid nutrition for diet protein reduction in poultry. J. Anim. Sci. Biotechnol..

[37] Salahuddin M., Abdel-Wareth A.A.A., Hiramatsu K., Tomberlin J.K., Luza D., Lohakare J. (2024). Flight toward sustainability in poultry nutrition with black soldier fly larvae. Animals.

[38] Ewald N., Vidakovic A., Langeland M., Kiessling A., Sampels S., Lalander C. (2020). Fatty acid composition of black soldier fly larvae (*Hermetia illucens*)—Possibilities and limitations for modification through diet. Waste Manag..

[39] Mwaniki Z.N., Neijat M., Kiarie E. (2018). Egg production and quality, fatty acid profile, and carotenoid content of eggs from laying hens fed full-fat insect meal. Poult. Sci..

[40] Nam J.-H., Hyun J.-Y., Lee B.K., Chun J.-Y. (2025). Effects of feeding mandarin and poultry by-products on growth performance and physicochemical properties of black soldier fly (*Hermetia illucens*) larvae. Food Sci. Anim. Resour..

